# How to Improve Urban Intelligent Traffic? A Case Study Using Traffic Signal Timing Optimization Model Based on Swarm Intelligence Algorithm

**DOI:** 10.3390/s21082631

**Published:** 2021-04-08

**Authors:** Xiancheng Fu, Hengqiang Gao, Hongjuan Cai, Zhihao Wang, Weiming Chen

**Affiliations:** 1 School of Mechanical Engineering and Electronic Information, China University of Geosciences, Wuhan 430074, China; fuxiancheng_cug@outlook.com; 2 School of Information Science and Engineering, Wuchang Shouyi University, Wuhan 430064, China; caihongjuan_cug@outlook.com; 3 Faculty of Engineering, China University of Geosciences, Wuhan 430074, China; wzh_gcxy@cug.edu.cn (Z.W.); chenweiming@cug.edu.cn (W.C.)

**Keywords:** intersection, traffic signal timing, labor division, real-time control

## Abstract

Traffic congestion is a major problem in today’s society, and the intersection, as an important hub of urban traffic, is one of the most common places to produce traffic congestion. To alleviate the phenomenon of congestion at urban traffic intersections and relieve the traffic pressure at intersections, this paper takes the traffic flow at intersections as the research object and adopts the swarm intelligent algorithm to establish an optimization model of intersection traffic signal timing, which takes the average delay time of vehicles, the average number of stops of vehicles and the traffic capacity as the evaluation indexes. This model adjusts the intersection traffic signal timing intelligence according to the real-time traffic flow and carries out simulation experiments with MATLAB. Compared with the traditional timing schemes, the average delay time of vehicles is reduced by 10.25%, the average number of stops of vehicles is reduced by 24.55%, and the total traffic capacity of the intersection is increased by 3.56%, which verifies that the scheme proposed in this paper is effective in relieving traffic congestion.

## 1. Introduction

The collective behavior of social insects through simple cooperation is called “swarming intelligence (SI)”. In 1992, Dorigo [1] proposed the ant colony algorithm (ACO) in the swarm intelligence algorithm. Then, Levy [2] first introduced swarm intelligence in detail in 1994. After that, particle swarm optimization (PSO) [3], artificial fish swarm algorithm (AFSA) [4], artificial bee colony algorithm (ABC) [5], and other algorithms based on biological foraging were proposed by scholars, and the research on swarm intelligence was gradually deepened. With the development of the information age, those algorithms have been widely used in various kinds of fields, including the public opinion polarization process [6,7,8,9], fall detection [10], analysis of user satisfaction [11], welding flame detection [12], and road networks [13,14].

Division of labor is also an important behavior of swarm intelligence [15], which has been widely used in task allocation [16,17], space allocation [18,19], robots, and Unmanned Aerial Vehicle (UAV) [20,21] in recent years.

In recent years, with the continuous improvement of people’s living standards, the traffic demand is also increasing, and traffic congestion has become a major problem faced by many cities. Because urban traffic is a complex system with many controlled variables [22], the urban traffic problem has become a “perennial difficulty” among many urban problems. For this reason, scholars have put forward various countermeasures and applied a variety of emerging technologies. Hakim et al. [23] proposed a fuzzy Technique for Order Preference by Similarity to an Ideal Solution (TOPSIS) algorithm based on the real-time number and classification of vehicles, which could set the priority vehicles according to the length of vehicles, and applied this algorithm in the design of the timing control system of traffic signal lights. Guo et al. [24] proposed a timing strategy including green time optimization and lane combination calculation. According to the real-time traffic flow, this strategy can optimize the greening time, calculate the lane combination, adjust the cycle, and get the timing plan. The experiment proves that this strategy can improve the traffic efficiency of the intersection by more than 15%. Chen et al. [25] proposed a traffic signal light control system based on model predictive control (MPC), which is divided into the traffic flow prediction model and the traffic timing optimization method. On the basis of the automatic collection of traffic flow data, appropriate time settings are obtained through the system to alleviate traffic congestion. Taking the cellular transmission model as the basic model, Qi [26] proposed an optimal timing model for a multi-intersection signal control scheme according to the characteristics of a multi-intersection road network, combined with control precision and real-time requirements. The model can adapt to the change of traffic flow at the intersection through time adjustment, and reduce the average delay time of road vehicles.

This paper will do some exploratory research on the application of the ant colony labor division model to solve the problem of traffic signal timing of the intersection. With taking the real-time traffic condition of the intersection as the research object, the optimization model of traffic signal timing of the intersection is established based on the evaluation index of the delay time of vehicles, the number of stops of vehicles, and the traffic capacity. This model is able to adjust the traffic signal timing intelligently according to real-time traffic conditions, reduce the conflict between traffic flows and alleviate the phenomenon of urban traffic congestion.

## 2. Ant Colony Labor Division Model

### 2.1. Basic Ant Colony Labor Division Model

Social insects will take the form of division of labor to complete tasks, and assign various tasks to different individuals. On the basis of an in-depth study of ant colony labor division behavior, Bonabeau et al. [27] proposed a fixed response threshold model, which believed that ant colony labor division behavior originated from the different responses of individual internal threshold to external stimuli. The stimulus value of the external task represents the urgency of the task, and the stimulus value varies with time and the number of ants currently performing the task. The internal threshold of an individual represents the tendency of the individual to perform the task, and the level of threshold is determined by the individual itself. When the stimulus value of the external task is far more than the internal threshold of the ant, the probability of the individual to perform the task is high. Otherwise, the probability of the individual to perform the task is low.

This paper uses *s* to represent the stimulus value of the external task, *θ* to represent the internal response threshold of an ant, Δ to represent the increase of the stimulus value of per unit time, α to represent the efficiency of the ant to perform the task, and *N_act_* to represent the number of ants currently performing tasks. Then, in each unit time, the probability of ants performing the task is shown in Equation (1), and the change of external task stimulus is shown in Equation (2). In the study of Castello et al. [28], *n* represents a constant that controls the curve shape of the individual response threshold function, which is generally set as *n* = 2.

(1) $$P=\frac{s^{n}}{s^{n}+\theta^{n}}$$

(2) $$\;s\left(t+1\right)=s\left(t\right)+\Delta -{\alpha N}_{\mathrm{act}}$$

### 2.2. Optimization of Ant Colony Labor Division Model

In the basic ant colony labor division model, ants will perform tasks in which environmental stimulus intensity exceeds their internal response threshold. In the traffic signal timing problem, different signal phases will increase or decrease the green time according to the traffic flow of each phase. Therefore, in the traffic signal timing problem, the signal phase can be regarded as different ants, and the traffic flow and green time of each phase can be regarded as the stimulus value of the external environment.

Here are the definitions of some of the notation involved in this model:

*q_i_*—the traffic flow at phase *i*;
*t_g_^i^*—the green time at phase *i*;
*s_i_*^+^—the stimulus value of increasing green time at phase *i*;
*s_i_*^−^—the stimulus value for the reduction of green time at phase *i*;
*θ_i_*^+^—the response threshold of increasing the green time in phase *i*;
*θ_i_*^−^—the response threshold of reducing the green time in phase *i*;
*P_i_*^+^—the selection probability of the green time increase in phase *i*;
*P_i_*^−^—the selection probability of the green time decrease in phase *i*;
P_i_^*^—the selection probability of the green time invariant in phase *i*;
*ξ_i_*^+^—the increment of green time for phase *i*;
*ξ_i_*^−^—the reduction of green time for phase *i*;
*λ_i_*—the green ratio in phase *i*;
*x_i_*—the effective green time of phase *i*;
*y_i_*—the degree of traffic flow saturation;
*C*—the cycle time;
*D_i_*—the delay time of vehicles in phase *i*;
*H_i_*—the number of stops of vehicles in phase *i*;
*Q_i_*—the traffic capacity in phase *i*.

#### 2.2.1. Environmental Stimulus

In the problem of green time allocation at a traffic intersection, if the total cycle time is fixed, when the green time of a certain phase increases, the traffic congestion at that phase is relieved, but it will inevitably lead to the decrease of green time of other phases and the increase of the probability of traffic congestion. Generally speaking, in the distribution problem, when the interest subjects increase their interests, their willingness to continue to increase will be relatively weakened, and the willingness to reduce the interests will be relatively enhanced. On the contrary, when the interest subject reduces their interests, their willingness to continue to reduce will be relatively weakened, and the willingness to increase the interests will be relatively enhanced.

Let the current traffic flow at phase *i* of the intersection be *q_i_* and the green time at phase i be *t_g_^i^*. The stimulus value of increasing green time at this phase is *s_i_^+^*, and the change of *s_i_^+^* is shown in Equation (3). As can be seen from Equation (3), when *t_g_^i^* remains constant, the higher *q_i_* is, the higher *s_i_^+^* is, indicating that the busier the current traffic flow is, the stronger the stimulus of increasing the green time of phase *i* is. Similarly, when *q_i_* remains constant, the lower *t_g_^i^* is, the higher *s_i_^+^* is, indicating that the shorter the current green time is, the stronger the stimulus of increasing the green time in phase *i* is.

(3) $$s_{i}^{+}=\frac{q_{i}}{q_{i}+t_{g}^{i}}$$

The stimulus value for the reduction of green time in phase *i* is *s_i_^−^*, and the change of *s_i_^−^* is shown in Equation (4). As can be seen from Equation (4), when *q_i_* remains unchanged, the higher *t_g_^i^* is, the higher *s_i_^−^* is, indicating that the longer the current green time is, the stronger the stimulus of reducing green time in phase *i* is. Similarly, when *t_g_^i^* remains unchanged, the lower *q_i_* is, the higher *s_i_^−^* is, indicating that the lower traffic flow is, the stronger the stimulus of reducing green time in phase *i* is.

(4) $$s_{i}^{-}=\frac{t_{g}^{i}}{q_{i}+t_{g}^{i}}$$

#### 2.2.2. Response Threshold

In the process of green time allocation at traffic intersections, the busier the traffic flow of a phase is, the greater the tendency of green time increase of the phase is. On the contrary, the smaller the traffic flow of a phase is, the greater the tendency to reduce the green time at this phase is, and the smaller the tendency to increase it is.

(5) $$\theta_{i}^{+}=\frac{1}{{\mathrm{kq}}_{i}}$$

(6) $${\;\theta }_{i}^{-}={\mathrm{kq}}_{i}$$

where *θ_i_^+^* is the response threshold of increasing the green time in phase *i*, and *θ_i_^−^* is the response threshold of reducing the green time in phase *i*.

Equations (5) and (6) show that *θ_i_^+^* is inversely proportional to *q_i_*. That is, when the traffic flow of phase *i* is busier, the response threshold of green time increase in this phase is lower, and the tendency of green time increase is greater. *θ_i_^−^* is proportional to *q_i_*. That is, when the traffic flow in phase *i* is lower, the response threshold of green time reduction in this phase is lower, and the tendency of green time reduction is greater.

#### 2.2.3. Probability of Task Execution

In the process of each time allocation, there are only three states for green lights of each phase: increase, constant, and decrease, and only one of them can be selected. The probability sum of the three states is 1.

(7) $$P_{i}^{+}=\frac{{\left(s_{i}^{+}\right)}^{2}}{{\left(s_{i}^{+}\right)}^{2}+{\left(\theta_{i}^{+}\right)}^{2}}=\frac{{\left(\frac{q_{i}}{q_{i}+t_{g}^{i}}\right)}^{2}}{{\left(\frac{C_{i}}{q_{i}+t_{g}^{i}}\right)}^{2}+{\left(\frac{1}{{\mathrm{kq}}_{i}}\right)}^{2}}$$

(8) $$P_{i}^{-}=\frac{{\left(s_{i}^{-}\right)}^{2}}{{\left(s_{i}^{-}\right)}^{2}+{\left(\theta_{i}^{-}\right)}^{2}}=\frac{{\left(\frac{t_{g}^{i}}{q_{i}+t_{g}^{i}}\right)}^{2}}{{\left(\frac{t_{g}^{i}}{q_{i}+t_{g}^{i}}\right)}^{2}+{\left({\mathrm{kq}}_{i}\right)}^{2}}$$

(9) $$P_{i}^{*}=1-P_{i}^{+}-P_{i}^{-}$$

where *P_i_^+^* is the selection probability of the green time increase in phase *i*, *P_i_^−^* is the selection probability of the green time decrease, and P_i_^*^ is the selection probability of the green time invariant.

Equations (7) and (8) show that the stronger the stimulus of increasing green time is, and the lower the response threshold of increasing green time is, the greater the probability of increasing green time is. Similarly, the stronger the stimulus of reducing green time is, and the lower the response threshold of reducing green time is, the greater the probability of green time reduction is.

#### 2.2.4. Task Response

In the ant colony labor division model, most tasks are expressed in the form of stimulus, and ants decide whether to perform tasks through probability. In the traffic intersection signal timing problem, the probability of task execution is used to determine whether the green time in a phase needs to be adjusted. Let *β* be the lower limit threshold of *P_i_*^+^. Only when *P_i_*^+^ is greater than *β*, i.e., *P_i_^+^* > *β* will the green time of the phase be increased. The increment is:

*ξ_i_*^+^ = *f*(*P_i_*^+^) (10)

In Equation (10), *ξ_i_^+^* is a positive correlation function of *P_i_^+^*. When the probability of green time increase is greater than the lower limit of the probability of green time increase, the green time of this phase increases. The greater the probability of task execution is, the more likely the green time will increase.

Let *α* be the lower limit threshold of *P_i_*^−^. Only when *P_i_*^−^ is greater than *α*, i.e., *P_i_*^−^ > *α*, will the green time of phase *i* be decreased. The reduction amount is:

*ξ_i_*^−^ = *f*(*P_i_*^−^) (11)

Similar to Equation (10), in Equation (11), *ξ_i_^−^* is a positive correlation function of *P_i_^−^*. When the probability of green time reduction is greater than the lower limit of the probability of green time reduction, the green time of the phase decreases. The greater the probability of task execution is, the more likely the green time will decrease.

When *P_i_^+^* <*β* and *P_i_^−^* < *α*, the green time of phase *i* remains unchanged.

#### 2.2.5. Algorithm implementation

**Figure 1:**
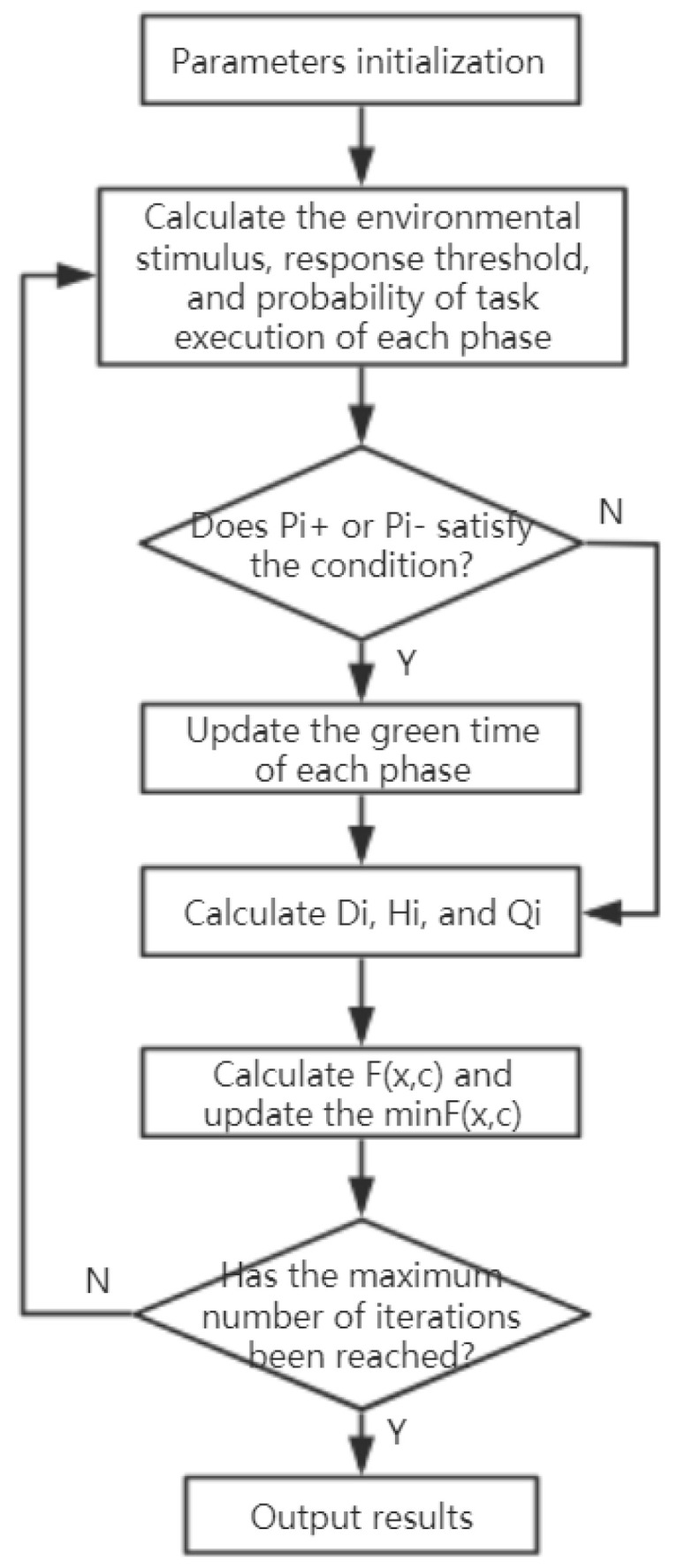
The algorithm calculation process.

According to the previous description of the model, the algorithm implementation process of the model is given, as shown in Figure 1. The process is described as follows:

(1) Parameters including phase number n, initial green time, scale coefficient k, maximum iteration number N, etc., are initialized.
(2) The initial number of iterations is set to 1.
(3) According to Equations (3)–(8), the environmental stimulus, response threshold, and probability of task execution of each phase are calculated.
(4) If *P_i_*^+^ is greater than *β* or *P_i_*^−^ is greater than *α*, jump to (5), otherwise, jump to (6).
(5) According to Equation (10) or (11), the change amount of green time is calculated and then the green time of each phase is updated.
(6) According to Equations (12)–(17), the delay time of vehicles *D_i_*, stopping times of vehicles *H_i_*, and the road traffic capacity *Q_i_* are calculated.
(7) The value of evaluation function *F*(*x*, *c*) is calculated according to Equation (18).
(8) Update the *min F*(*x,c*), and the corresponding optimal solution of green time.
(9) Increase the number of iterations by 1.
(10) Judge whether the maximum number of iterations has been reached. If so, jump to (11); otherwise, jump to (3).
(11) Output the optimal solution.

## 3. Experiment and Analysis

### 3.1. Design of the Signal Phase

**Figure 2:**
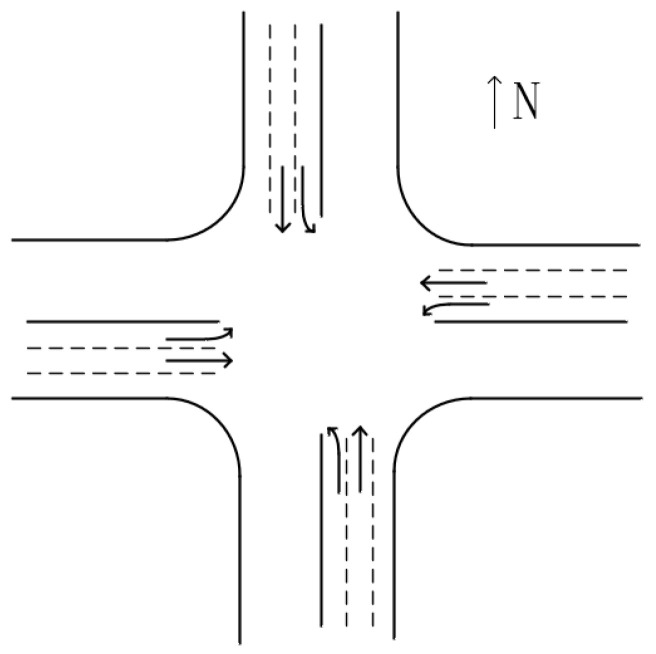
A crossroad.

There are many types of intersections in the city, including intersection, roundabout, and interchange. Intersection includes T-shaped intersection, Y-shaped intersection, X-shaped intersection, cross-shaped intersection, and so on. For the sake of simplification, only the most common cross-shaped intersections in daily life are considered in this paper, of which shapes are shown in Figure 2. The intersection points to the different directions of the road, and the vehicles drive in different directions, which leads to the intersection become a traffic accident-prone point, known as the conflict point [29]. In Figure 2, the east, west, south, and north directions of the intersection are divided into left, straight and right lanes, and vehicles drive in the corresponding lanes.

**Figure 3:**
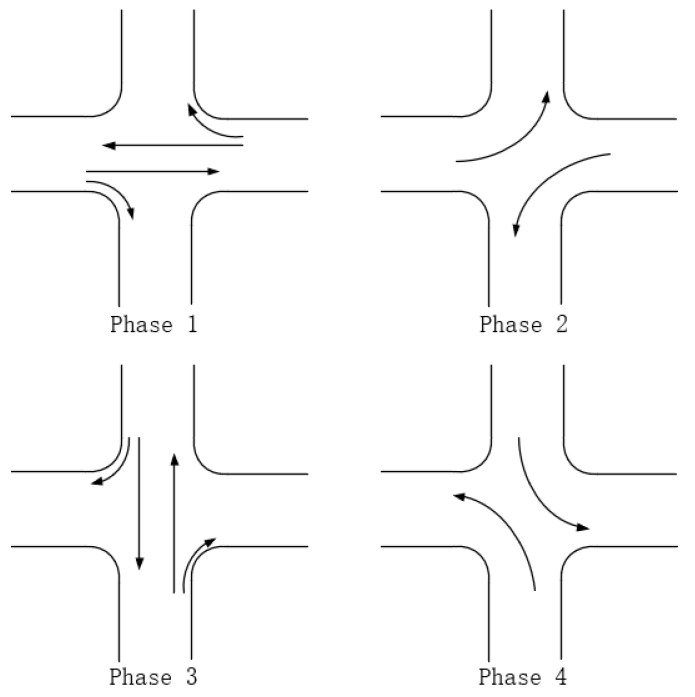
The four-phase scheme.

The phase scheme of the traffic signal lamp includes the two-phase scheme, the three-phase scheme, the four-phase scheme and so on. In the four-phase scheme, the special left-turn phase is set in each direction. Compared with the two-phase scheme and the three-phase scheme, the conflict points are effectively eliminated and the traffic efficiency is improved when the road is busy, so this paper discusses the four-phase scheme, and its schematic diagram is shown in Figure 3.

### 3.2. Experiment Data

**Figure 4:**
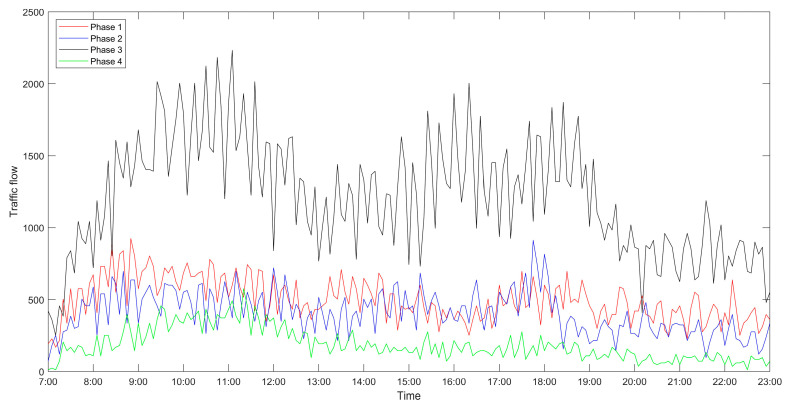
Traffic flow at the intersection of Wuhe Avenue and Zhangheng Road.

The data used in this paper come from “2020 China University computer competition · Huawei cloud big data challenge”, which describes the traffic situation at the intersection of Wuhe Avenue and Zhangheng Road in Longgang, Shenzhen. It intercepts the traffic flow at the intersection from 7 am to 11 pm on 12 January, 2020, and sets every five minutes as a unit time. Due to the excessive number of straight cars in all directions of the intersection, in order to eliminate the conflict points and reduce the possibility of accidents, the four-phase scheme is selected. After processing, the traffic flow at the intersection on that day is shown in Figure 4.

### 3.3. Evaluation Index

Under the premise of ensuring traffic safety, the main purpose of signal timing at the intersection is to maximize the efficiency of the intersection, not only to make full use of road traffic resources, but also to protect the rights and interests of road users. Therefore, the average delay time of vehicles, the average number of stops of vehicles and the traffic capacity are selected as evaluation indexes to optimize the traditional traffic signal timing scheme.

For intersections, the vehicle delay formula on the Webster algorithm has been widely used in the field of traffic control [30]. The specific formulas are as follows [31]:

(12) $$D_{i}=\frac{C{\left(1-\lambda_{i}\right)}^{2}}{2\left(1-\lambda_{i}y_{i}\right)}+\frac{y_{i}^{2}}{2q_{i}\left(1-y_{i}\right)}$$

(13) $$T=\frac{\sum_{i=1}^{n}D_{i}q_{i}}{\sum_{i=1}^{n}q_{i}}$$

where *D_i_* is the delay time of vehicles in phase *i*, *T* is the average delay time of vehicles in a cycle, *C* is the total duration of the cycle, *λ_i_* is the green ratio which is the ratio of the effective green time of phase i to the total duration of the cycle, *y_i_* is the degree of traffic flow saturation which is the traffic flow in phase *i* to the saturated traffic flow, *q_i_* is the traffic flow in phase *i*.

(14) $$H_{i}=0.9\frac{1-\lambda_{i}}{1-y_{i}}$$

where *H_i_* is the number of stops of vehicles in phase *i*.

(15) $$H=\frac{\sum_{i=1}^{n}H_{i}q_{i}}{\sum_{i=1}^{n}q_{i}}$$

where *H* is the average number of stops of vehicles in phase *i*.

(16) $$Q_{i}=S_{i}\lambda_{i}=S_{i}\frac{x_{i}}{C}\;$$

where *Q_i_* [31] is the traffic capacity in phase *i*, *S_i_* is the traffic saturation flow rate in phase *i*, *x_i_* is the effective green time of phase *i*.

(17) $$Q=\overset{n}{\underset{i}{\sum }}Q_{i}\;$$

where *Q* [29] is the total traffic capacity of the intersection.

### 3.4. Experiment

The traffic signal timing scheme proposed in this paper will be compared with the traditional fixed timing scheme. In the traditional fixed timing scheme, the cycle length and the green time of each phase in a day remain unchanged. The scheme proposed in this paper will judge whether the cycle length and green time should be adjusted according to the number of vehicles passing through the intersection. The solution optimization model is given in reference [32]:

(18) $$\min F\left(x,c\right)=\frac{\sum_{i=1}^{n}\left(K_{1}D_{i}+K_{2}H_{i}\right)}{\sum_{i=1}^{n}K_{3}Q_{i}}$$

It is assumed that the saturated traffic flow at each phase of the intersection is 1000 pcu/h, 1000 pcu/h, 2500 pcu/h and 600 pcu/h. The time of the amber is 3 s, the change interval is 5 s, and the start-up delay is 3 s. The shortest and longest green time is 20 s and 90 s, and the initial green time is 40 s. *f*(*Pi*^+^) is an exponential function with e as the base and Pi^+^ as the exponent. *f*(*Pi*^−^) is an exponential function with e as the base and *Pi*^−^ as the exponent. The maximum number of iterations *N* is set to 50. The simulation calculation environment is described as follows: MATLAB language is used to complete the implementation of the algorithm in this paper, and the experiment is carried out on a PC with a 2.5 GHz CPU and 8 GB memory.

**Figure 5:**
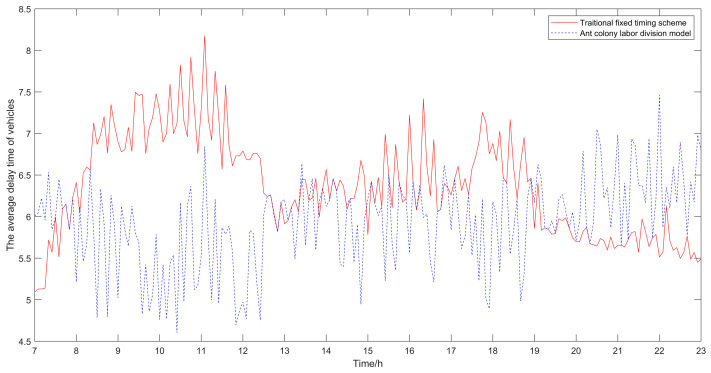
Comparison diagram of average vehicle delay time.

**Figure 6:**
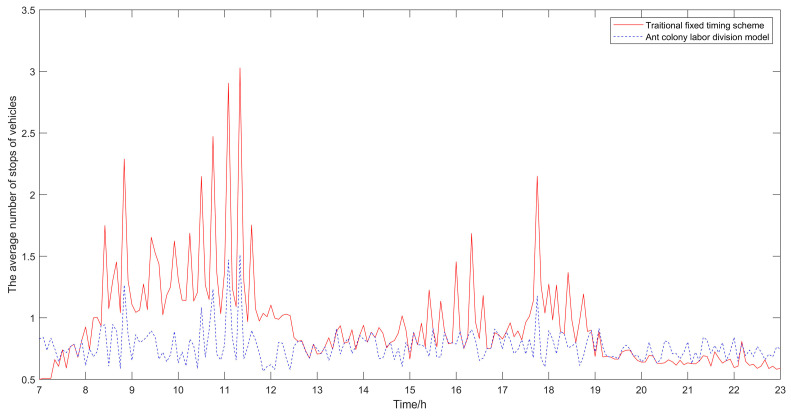
Comparison diagram of the average number of stops of vehicles.

**Figure 7:**
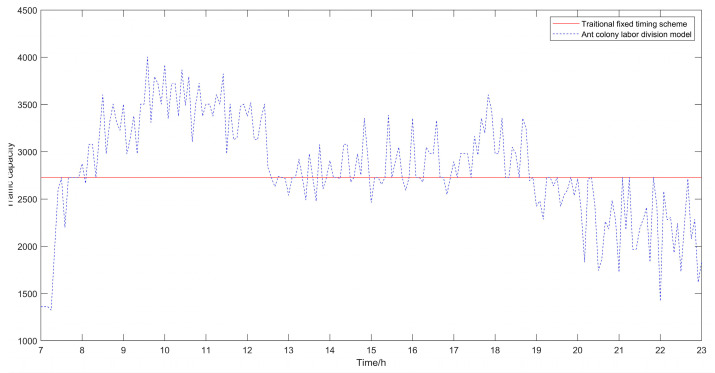
Comparison diagram of traffic capacity at intersections.

The algorithm took 1.99 s to execute. The results of the traditional fixed timing scheme and the optimized results using the ant colony labor division model proposed in this paper are shown in Table 1, Table 2 and Table 3. The result comparison diagrams are shown in Figure 5, Figure 6 and Figure 7.

### 3.5. Result Analysis

It can be seen from Table 1, Table 2 and Table 3 that compared with the traditional fixed timing scheme, the scheme optimized by the ant colony labor division model has significant improvement in the delay time of vehicles and the number of stops. The average delay time of vehicles in the traditional fixed timing scheme is 6.53 s, and in the optimized scheme is 5.86 s, which is 10.25% lower than that in the traditional fixed-timing scheme. The average number of stops in the traditional fixed timing scheme is 1.0314, and after optimization, it is 0.7782, which is 24.55% lower than that in the traditional fixed timing scheme. It can be seen that in terms of the average delay time of vehicles and the average number of stops, the results of the proposed optimization algorithm proposed in this paper are obviously better than those of the traditional fixed timing scheme.

Besides, the results of the optimized algorithm also have a corresponding improvement in the traffic capacity, but the improvement is not significant. In order to better analyze the changes before and after optimization, the comparison diagrams of the average vehicle delay time, the average number of stops of vehicles and the traffic capacity at intersections are shown (as shown in Figure 4, Figure 5 and Figure 6). As can be seen from Figure 6, the traffic capacity has been greatly improved during 9:00–12:00 on that day, and from 14:00 to 19:00. Although the data fluctuates up and down, the traffic capacity is improved. The traffic capacity, however, decreased during 7:00–8:00 and 19:00–23:00. Combined with Equations (16) and (17), and the trend analysis of traffic flow in Figure 3, it is found that the traffic capacity of the optimized algorithm is related to the effective green time of the phase. When the traffic flow of this phase is very low, according to the algorithm, the green time of this phase will be decreased. Correspondingly, the effective green time will also be decreased, and the green ratio will be decreased, leading to the decrease of the traffic capacity of the intersection. However, from the point of the traffic flow saturation in the intersection, there is no over-saturation state in the whole day, nor does it exceed the maximum capacity of the intersection. It can be said that this phenomenon has very little impact on the traffic operation efficiency of the intersection.

From Figure 5, Figure 6 and Figure 7, it can be seen that in the period of busy traffic flow, such as 8:00–12:00 and 17:00–19:00, the average delay time and the average number of stops of vehicles after optimization are significantly reduced, and the intersection capacity is also significantly improved. According to calculation, during the period of 8:00–12:00, compared with the fixed timing scheme, the average delay time of vehicles is reduced by 22.01% in the timing scheme optimized by the ant colony labor division model, the average number of stops of vehicles is reduced by 40.92%, and the road capacity is increased by 24.37%. During the period of 17:00–19:00, the average delay time of vehicles is reduced by 12.46%, the average number of stops of vehicles is reduced by 25.29%, and the road capacity is increased by 9.92%. However, in the period after 20:00, due to the small traffic flow, the possibility of congestion is low, and the optimization effect of the algorithm is not obvious.

In general, the scheme proposed in this paper is more suitable for the situation of heavy traffic flow, and the greater the traffic flow, the better the improvement effect of the scheme on vehicle delay time, number of stops and road capacity will be. Ant colony labor division model can alleviate traffic congestion at intersections to a certain extent and improve the utilization rate of roads.

## 4. Conclusions

In the problem of traffic intersection green time allocation, the traffic flow in each phase changes dynamically. In order to relieve the congestion at the intersection, the traffic signal green time of each phase should change dynamically with the traffic flow, rather than remain unchanged.

As a new optimization model, the ant colony labor division model has been widely applied to the dynamic task assignment problem because of its flexibility. In this paper, the ant colony labor division model is applied to the problem of traffic green time allocation at traffic intersections. The simulation experiment results show that compared with the fixed timing scheme, the proposed timing scheme can reduce the average delay time of vehicles by 10.25% and the average number of stops by 24.55%, and it can increase the road capacity by 3.56%. These data proved that the effect of this scheme is better than the traditional fixed timing scheme, and it can better alleviate the traffic pressure at the intersections during rush hours.

Due to the strong real-time uncontrollability of the traffic situation at the intersection, the next research direction is to further optimize the precision of the model, and try to combine it with the video monitoring, which can obtain the traffic flow and other information, as well as extend the application range from the single intersection to urban trunk lines, and even extended to larger urban traffic network.

## Figures and Tables

**Table 1:** Comparison of the average delay time of vehicles at intersections.

Phase i	Traditional Fixed Timing Scheme/s	Ant Colony Labor Division Model/s	Relative Change/%
Phase 1	6.65	6.04	−9.06
Phase 2	6.27	5.59	−10.71
Phase 3	6.63	5.97	−9.95
Phase 4	6.15	5.23	−14.83
The average of the intersections	6.53	5.86	−10.25

**Table 2:** Comparison of the average number of stops of vehicles at intersections.

Phase i	Traditional Fixed Timing Scheme	Ant Colony Labor Division Model	Relative Change/%
Phase 1	1.0780	0.8253	−23.44
Phase 2	0.8871	0.6885	−22.38
Phase 3	1.0683	0.8071	−24.44
Phase 4	0.9831	0.6602	−32.84
The average of the intersections	1.0314	0.7782	−24.55

**Table 3:** Comparison of traffic capacity at intersections.

Phase i	Traditional Fixed Timing Scheme/pch·h^−1^	Ant Colony Labor Division Model/pch·h^−1^	Relative Change/%
Phase 1	6073	6329	+4.23
Phase 2	6073	6252	+2.95
Phase 3	15,181	15,832	+4.28
Phase 4	3644	3660	+0.45
The intersection traffic capacity	30,969.76	32,072.55	+3.56

## Data Availability

The study used the open data.
